# Causal effects of autoimmune diseases on thyroid cancer: a two-sample Mendelian randomization study

**DOI:** 10.3389/fendo.2024.1401458

**Published:** 2024-08-08

**Authors:** Wenfang Peng, Bojin Xu, Haiping Zhou, Juan Du, Xiaoxu Ge, Shan Huang

**Affiliations:** Department of Endocrinology, Tongren Hospital, Shanghai Jiao Tong University School of Medicine, Shanghai, China

**Keywords:** autoimmune disease, thyroid cancer, Mendelian randomization, causality, systemic lupus erythematosus, primary biliary cirrhosis

## Abstract

**Background:**

Although numerous studies had revealed associations between autoimmune diseases (AIDs) and thyroid cancer (TC), the potential causal associations between the two remain poorly defined.

**Methods:**

Using five approaches, two-sample Mendelian randomization (MR) analyses were carried out to determine the causal effects of 12 major AIDs on risk of TC. The sensitivity analyses were conducted to verify the reliability of the analysis. The reverse MR analysis was performed to evaluate the possibility of reverse causation.

**Results:**

The results showed a significant causal association of systemic lupus erythematosus (SLE) and primary biliary cirrhosis (PBC) on the risk of TC. Genetically predicted PBC elevated the risk of TC (OR = 1.46, 95% CI = 1.06-2.02, *p* = 0.021). The risk of TC was also increased by genetically predicted SLE (OR = 6.52, 95% CI = 1.38-30.84, *p* = 0.018) with heterogeneity. After outlier-corrected analyses, the results still suggested that genetically predicted SLE increased the risk of TC (*p* = 0.019). No evidence of a causal relationship between the remaining 10 AIDs and TC was observed. No reverse causal effects of TC on AIDs were found in reverse MR analysis.

**Conclusion:**

These findings support a significant causal association of SLE/PBC on the increased risk of TC, indicating that patients with SLE/PBC should be under a close monitoring of TC.

## 1 Introduction

Thyroid cancer (TC) is a prevalent endocrine malignant tumor in China, with an estimated number of new cases exceeding 224,000 in 2022 (1). TC arises from endoderm-derived follicular cells or neural crest-derived C-cells and comprises papillary, follicular, medullary, and undifferentiated subtypes (2). With the increasing use of diagnostic imaging and surveillance, its incidence continues to rise steadily worldwide (3, 4). The precise causes of TC are multifactorial, complicated, and not well understood. Apart from the most-established risk factor for TC (childhood exposure to ionizing radiation), other possible risk factors such as estrogen, chromosomal and genetic alterations, lifestyle changes, autoimmune thyroid disease, and excess body weight have been reported (5). Converging lines of evidence indicate that the immune system exerts momentous roles during the multistep development of TC (6–8).

Autoimmune disease (AID) is an excessive host immune response with diverse clinical manifestations caused by the impairment of the host immune regulatory function (9). As we all know, both cancers and AIDs share the feature of immune system dysregulation. Accumulating evidence reveals that AIDs have been associated with multiple tumors. Zeev Elkoshi suggested that pre-existing AIDs promoted the initiation of cancer and its early growth, leading to an increased risk of cancer (10). A review indicates that the majority of autoimmune rheumatic diseases are linked to a slightly elevated risk of malignancy (11). A significant association to develop cancer in scleroderma patients has been observed when compared with the general population (12). Evidence supporting the intimate relationship between dermatomyositis and malignancies, such as non-Hodgkin lymphoma, lung, and colorectal cancers, has emerged (13). Hemminki et al. suggested that the autoimmune process in AIDs contributed to lung cancer susceptibility (14). Yang et al. indicated an association between Sjogren’s syndrome and increased risks of head and neck cancers, encompassing several sites including the oral cavity, oropharynx, nasopharynx, and thyroid (15). Chen et al. reported that patients with Graves’ disease (GD) exhibited a significantly higher risk of developing TC, with a 16-fold hazard within the first three years of diagnosis compared to non-GD patients (16). Due to the inherent limitations of observational studies, such as reporting biases and potential confounding factors, it cannot be proven whether there is a causal relationship between AIDs and TC.

By employing genetic variants as instrument variables (IVs), Mendelian randomization (MR) could evaluate the causality between an exposure and outcome (17). In this work, the two-sample MR analysis was employed to deduce the causative relationships between 12 major AIDs, including systemic lupus erythematosus (SLE), ankylosing spondylitis (AS), rheumatoid arthritis (RA), primary biliary cirrhosis (PBC), multiple sclerosis (MS), ulcerative colitis (UC), type 1 diabetes (T1D), celiac disease (CeD), Crohn’s disease (CD), asthma, psoriasis (PsO), and hypothyroidism, and the risk of TC using summary data from genome-wide association studies (GWAS). Moreover, a reverse MR analysis was conducted to investigate the potential for reverse causality.

## 2 Materials and methods

### 2.1 Data sources

To infer causal relationships between TC and AIDs, GWAS summary statistics on TC and 12 AIDs derived from the IEU OpenGWAS project (https://gwas.mrcieu.ac.uk) were downloaded. All individuals involved in this study were of European ancestry. **Table 1** presented the specific data sources in detail.

**Table 1:** Details of data sources.

Disease	GWAS ID	Sample	Case	Control	SNP	Population	Author
Systemic lupus erythematosus	ebi-a-GCST003156	14267	5201	9066	7071163	European	Bentham J
Rheumatoid arthritis	ebi-a-GCST90013534	58284	14361	43923	13108512	European	Ha E
Multiple sclerosis	ebi-a-GCST005531	38582	14498	24091	132089	European	Beecham AH
Crohn’s disease	ebi-a-GCST003044	20883	5956	14927	110583	European	Liu JZ
Ulcerative colitis	ieu-a-970	47745	13768	33977	156116	European	Liu
Ankylosing spondylitis	ebi-a-GCST005529	22647	9069	1550	99962	European	Cortes A
Type 1 diabetes	ebi-a-GCST010681	24840	9266	15574	12783129	European	Forgetta V
Primary biliary cirrhosis	ebi-a-GCST005581	11375	2861	8514	119756	European	Liu JZ
Psoriasis	ebi-a-GCST005527	33394	10588	22806	138661	European	Tsoi LC
Celiac disease	ebi-a-GCST000612	15283	4533	10750	518292	European	Dubois PC
Asthma	ebi-a-GCST90014325	408442	56167	352255	34551291	European	Valette K
Hypothyroidism	ebi-a-GCST90018862	410141	30155	379986	24138872	European	Sakaue S
Thyroid cancer	ieu-a-1082	1080	649	431	572028	European	Kohler A

GWAS, genome-wide association study; SNP, single nucleotide polymorphism.

### 2.2 Single nucleotide polymorphism selection

SNPs were determined as the IVs with genome-wide significance (*p <*5×10^-8^). Linkage disequilibrium clumping was performed by setting r^2^ <0.001 and clump distance = 10,000 kb, ensuring the independence of SNPs associated with exposure. The *F-statistic* was calculated to determine instrument strength using the formula: F = (β_exposure_/SE_exposure_)^2^. The *F-statistic* larger than 10 indicates a slight possibility of a weak IV deviation.

### 2.3 Study design

In order to accurately infer potential causal relationships between TC and AIDs, 3 assumptions should be met: (1) IVs are significantly associated with the exposure; (2) IVs are independent of any confounding factor; and (3) IVs affect the outcomes exclusively via exposure instead of through any alternative pathway (18) (**Supplementary Figure S1**). The process of this study was carried out in strict accordance with the Strengthening the Reporting of Observational Studies in Epidemiology Using Mendelian Randomization (STROBE-MR) Statement.

### 2.4 Statistical analysis

Five MR analysis methods based on different assumptions, including simple mode, inverse-variance-weighted (IVW), MR-Egger regression, weighted median (WM), and weighted mode, were applied to estimate the causal associations. IVW, as the main analysis method, combines the Wald ratio of each SNP to provide consistent causal estimates of the exposure on the outcome. The heterogeneity was assessed by Cochran’s Q test. The WM method offers unbiased causal estimates when the valid IVs account for more than half of the weight. The horizontal pleiotropy was evaluated with the MR-Egger method. We used the simple mode and weighted mode methods to supplement IVW estimates. The detection of outlier IVs and correction of horizontal pleiotropy were achieved by the MR-Pleiotropy Residual Sum and Outlier (MR-PRESSO) method. “TwoSampleMR” (version 0.5.8) and “MRPRESSO” (version 1.0) packages in R software (version 4.2.3) were employed to conduct MR analyses.

## 3 Results

### 3.1 IVs selection

The IVs that were significantly associated with 12 AIDs were obtained from the GWAS. Then, palindromic SNPs (ie., A/T or G/C) and SNPs that were not present in the outcome were eliminated. Finally, 8 IVs for SLE, 16 IVs for RA, 17 IVs for MS, 44 IVs for CD, 30 IVs for UC, 9 IVs for AS, 4 IVs for T1D, 5 IVs for PBC, 8 IVs for PsO, 7 IVs for CeD, 7 IVs for asthma, and 18 IVs for hypothyroidism (*F-statistic* > 10) were selected.

### 3.2 Causal effects of AIDs on TC

**Figure 1:**
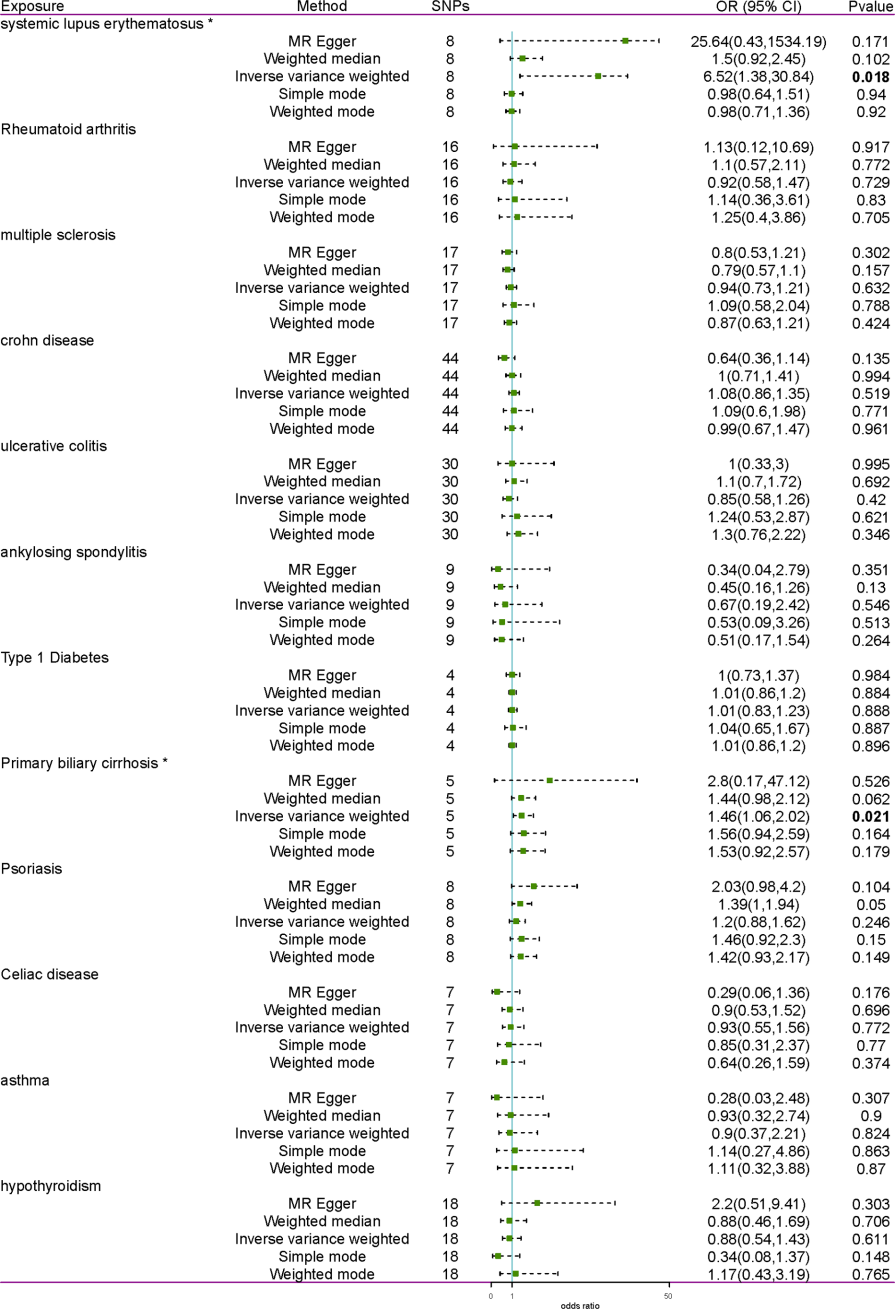
Forest plot of the effect of AIDs on TC. AID, autoimmune disease; TC, thyroid cancer; SNP, single- nucleotide polymorphism; OR, odds ratio. * indicates inverse variance weighted result with p <0.05. Results with a p <0.05 are bolded.

The MR analyses suggested a significant causal association of SLE and PBC on the risk of TC (**Figure 1**). With the IVW method, genetically predicted PBC elevated the risk of TC (OR = 1.46, 95% CI = 1.06-2.02, *p* = 0.021). Cochran’s Q test revealed no heterogeneity (MR-Egger Cochran’s Q = 0.518, *p* = 0.915; IVW Cochran’s Q = 0.725, *p* = 0.948) (**Table 2**). The risk of TC was likewise increased by genetically predicted SLE (OR = 6.52, 95% CI = 1.38-30.84, *p* = 0.018). Nonetheless, considerable heterogeneity was found in IVW (Cochran’s Q = 412.75, *p* = 4.393E-85) and MR-Egger (Cochran’s Q = 412.75, *p* = 4.393E-85). Then, the random effects IVW method was performed to assess the causal relationships. After removing 6 outliers (rs1143679, rs13019891, rs2431697, rs2459611, rs7097397, and rs7823055) detected by the MR-PRESSO test, only two SNPs remained. Hence, SNPs with genome-wide significance (*p <*5×10^-6^) were determined as the IVs. After eliminating linkage disequilibrium and deleting palindromic SNPs and SNPs not available in the outcome, a total of 12 SNPs were obtained. The IVW method was performed to estimate causality again, indicating a significant causal relationship between SLE and TC (*p* = 0.004). After MR-PRESSO detecting outliers, the results indicated that the risk of TC was elevated by genetically predicted SLE (*p* = 0.019).

**Table 2:** Pleiotropy and heterogeneity test of the MR analysis.

Exposure	Heterogeneity test						Pleiotropy test		
	MR Egger			Inverse variance weighted					
	Q	Q_df	Q_p-value	Q	Q_df	Q_p-value	Egger intercept	se	p-value
Systemic lupus erythematosus	380.437	6	4.478E-79	412.750	7	4.393E-85	-0.526	0.740	0.502
Rheumatoid arthritis	13.222	14	0.509	13.255	15	0.583	-0.02	0.111	0.858
Multiple sclerosis	16.821	15	0.330	17.907	16	0.329	0.038	0.039	0.341
Crohn’s disease	43.198	42	0.420	47.023	43	0.311	0.072	0.038	0.061
Ulcerative colitis	49.773	28	0.007	49.930	29	0.009	-0.018	0.062	0.769
Ankylosing spondylitis	15.126	7	0.034	16.528	8	0.035	0.060	0.075	0.447
Type 1 diabetes	4.367	2	0.113	4.389	3	0.222	0.009	0.092	0.928
Primary biliary cirrhosis	0.518	3	0.915	0.725	4	0.948	-0.162	0.357	0.680
Psoriasis	6.579	6	0.362	9.202	7	0.238	-0.15	0.097	0.173
Celiac disease	8.906	5	0.113	13.184	6	0.040	0.270	0.174	0.182
Asthma	3.013	5	0.698	4.332	6	0.632	0.105	0.091	0.303
Hypothyroidism	19.520	16	0.243	21.604	17	0.200	-0.093	0.071	0.210

MR, Mendelian randomization.

Except for SLE and PBC, the MR analyses showed no causal association between the remaining 10 AIDs and TC. Significant heterogeneity was detected by Cochran’s Q test in both the MR-Egger and IVW methods of UC and AS and the IVW method of CeD (**Table 2**). Then, the random effects IVW method was performed again, indicating no causal association between UC, AS, CeD, and TC. The MR-Egger intercept test revealed no horizontal pleiotropy (*p >*0.05). Furthermore, leave-one-out analysis revealed that no single SNP can strongly affect the overall causal effect of AIDs on TC (**Supplementary Figure S2**). In summary, the MR analyses showed that SLE and PBC were substantially linked to an elevated risk of TC after sensitivity and pleiotropy analyses at the Bonferroni-corrected significance level (*p <*0.05).

### 3.3 Causal effects of TC on AIDs

**Figure 2:**
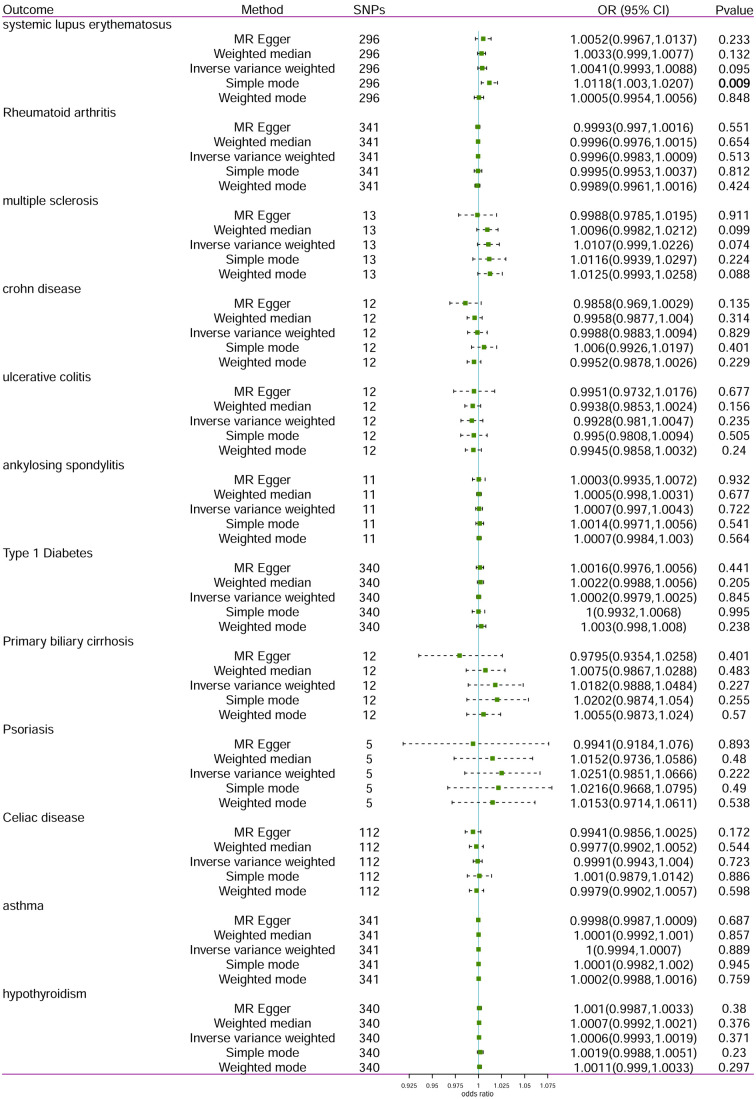
Forest plot of the effect of TC on AIDs. TC, thyroid cancer; AID, autoimmune disease; SNP, single- nucleotide polymorphism; OR, odds ratio. Results with a p <0.05 are bolded.

The reverse MR analysis was conducted to determine whether TC has any causal effect on AIDs using SNPs associated with TC as IVs. The reverse MR analysis used the identical IV selection criteria as the primary MR analysis. All MR approaches indicated that there was no reverse causal relationship between TC and AIDs with partial heterogeneity (**Figure 2**, **Table 3**). After MR-PRESSO detecting outliers, the results still support that there was no reverse causal relationships between TC and AIDs. In reverse MR analysis, the MR-Egger intercept test (*p >*0.05) showed no horizontal pleiotropy.

**Table 3:** Pleiotropy and heterogeneity test of the reverse MR analysis.

Outcome	Heterogeneity test						Pleiotropy test		
	MR Egger			Inverse variance weighted					
	Q	Q_df	Q_p-value	Q	Q_df	Q_p-value	Egger intercept	se	p-value
Systemic lupus erythematosus	921.760	294	6.62E-66	922.060	295	1.06E-65	-0.002	0.005	0.757
Rheumatoid arthritis	366.406	339	0.147	366.486	340	0.155	0.000	0.001	0.786
Multiple sclerosis	20.790	11	0.036	24.260	12	0.019	0.018	0.013	0.203
Crohn’s disease	23.518	10	0.009	31.163	11	0.001	0.018	0.010	0.102
Ulcerative colitis	36.834	10	6.05E-05	37.063	11	0.000	-0.003	0.013	0.808
Ankylosing spondylitis	37.056	9	2.57E-05	37.115	10	5.40E-05	0.001	0.005	0.907
Type 1 diabetes	366.824	338	0.135	367.525	339	0.138	-0.002	0.003	0.422
Primary biliary cirrhosis	24.510	10	0.006	34.189	11	0.0003	0.052	0.026	0.075
Psoriasis	0.848	3	0.838	1.619	4	0.805	0.032	0.037	0.445
Celiac disease	112.013	110	0.429	114.072	111	0.402	0.007	0.005	0.158
Asthma	363.135	339	0.176	363.506	340	0.182	0.000	0.001	0.557
Hypothyroidism	695.921	338	5.51E-27	696.335	339	7.13E-27	-0.001	0.001	0.654

MR, Mendelian randomization.

## 4 Discussion

AIDs are characterized by immune dysregulation and reactivity to self-antigens, resulting in immune-mediated destruction of own cells and tissues, which further leads to tissue damage and dysfunction (19). Due to the importance of immune system in recognition and elimination of tumors, immune system dysregulation is thought to increase the risk of cancer (20). Here, we performed the first two-sample MR analyses to determine the causal relationships between AIDs and TC. The MR analyses support a significant causal association of SLE and PBC on the risk of TC. However, no apparent genetic causal relationship between ten other AIDs (RA, MS, CD, UC, AS, T1D, PsO, CeD, asthma, and hypothyroidism) and TC was observed. The reverse MR analysis demonstrates that genetically predicted TC had no causal effect on AIDs (*p >*0.05).

Research on the association between SLE and cancer has been conducted for decades. The risk of prostate cancer was decreased in SLE patients, which may possibly be due to the low hypoadrenergic states that may occur in men with SLE (21). Bernatsky et al. reported a decreased risk of breast, ovarian, and endometrial cancers in SLE (22). The association between SLE and a small overall increased risk of certain cancers had been reported as well (23). A review based on the evidence from a meta-analysis revealed that SLE patients are at increased risk of developing lung, bladder, and liver cancers (24). In comparison to age-matched controls, SLE patients showed a higher prevalence of papillary TC in a case-control study (25). A review summarizes the scientific literature on the association between SLE and TC (26). A meta-analysis indicated a positive association between TC and SLE risk and concluded that patients with SLE had an increased risk of developing TC (27). Our results from MR analyses also support that genetically predicted SLE increases the risk of TC, indicating that patients with SLE should be under close surveillance for TC.

Increasing studies have indicated that PBC may be related to the risk of some cancers. Hepatocellular carcinoma (HCC) has been reported to be a potentially fatal complication for patients with PBC (28). HCC is not rare in Chinese PBC patients, with a significantly higher incidence in males than in females (29). A meta-analysis indicated that PBC patients had a higher risk of both HCC and overall cancer (30). Another study reported that genetically predisposed PBC reduced the risk of developing gastric cancer (31). Up to date, there are no studies linking PBC with TC. Our study is the first to support a significant causal association between PBC and TC, and patients with PBC are more vulnerable to developing TC, which may be a clinical issue worthy of contemplation.

The association between inflammatory bowel diseases (CD and UC) and TC risk has been reported but is controversial. The case-control study in the United States reported that patients with CD, not UC, were associated with a higher risk for TC than those with diverticulitis (32). Conversely, the meta-analysis performed by Lihong Cao suggested no association between CD and the increased risk of TC, and UC was related to the elevated risk of TC (33). The population-based cohort study from China showed that digestive cancers, TC, and hematological malignancies were the top three highest incidence cancers in UC patients, and no evidence supported the association between CD and cancer risk (34). In the present study, the MR analyses show no causal association between UC/CD and the risk of TC. This inconsistency may be caused by differences in genetic, environmental, or other confounding factors due to population differences in the study cohorts.

A large-scale cohort study in China indicated an increasing risk of developing TC in RA patients (35). A nationwide cohort study in Korea suggested a significantly elevated risk of TC in patients with RA compared with the control group without AIDs (36). A longitudinal analysis based on the Korean population exhibited higher odds of TC for male RA patients in the sex-stratified subgroup analyses (37). However, another early study in Korea reported that women patients with RA showed increased risks of TC (38). The nested case-control study in Korea reported that PsO was unrelated to the risk of TC in the overall adult population (39). In contrast, another cohort study, also in Korea, indicated that the TC risk was greater in patients with PsO than in those without PsO (40). In the nested case-control study in Finland, an elevated incidence of TC was observed in MS patients during the disease modifying treatment era (41). The nationwide cohort study in Taiwan indicated that patients with AS were at an increased risk of TC (42). The nationwide cohort of patients in the Swedish population showed that patients with CeD had no increased risk of TC, which was different from studies in Italy and the United States (43). A meta-analysis of cohort study reported that patients with diabetes mellitus were at increased risk of TC (44). Mäkimattila et al. indicated a marginally increased risk of TC in individuals with T1D compared to control individuals (45). The association between hypothyroidism and TC had been reported, with a higher prevalence of TC in patients with hypothyroidism (46). In addition, there is no study to describe the association between asthma and TC. Our findings showed no causal relationship between these AIDs and TC. Associations between these AIDs and TC found in previous observational research might be mediated by hitherto unknown confounding variables.

In conclusion, this is the first two-sample MR study to estimate the causality between AIDs and TC, which may offer more precise recommendations for TC monitoring in patients with AIDs. The results support a significant causal association of SLE and PBC on the risk of TC and do not support an association of the remaining ten AIDs with TC risk. It’s worth noting that the relationships between the remaining ten AIDs and TC risk may be weaker or require further exploration through extensive cohort studies. In addition, the reverse MR analyses demonstrate that genetically predicted TC has no causal effect on AIDs. However, the study population enrolled in the MR analyses was mainly from Europe, limiting the generalizability of our findings to individuals of other ethnicities. Importantly, these findings are based on predictions derived from genetic associations and should be considered preliminary. While our study provides statistical evidence for the causal associations between AIDs and TC, further research is needed to confirm these relationships and unravel the underlying mechanisms. Specifically, the connection between SLE, PBC, and TC risk requires additional confirmation. Furthermore, owing to limitations in the database, we were unable to delve into other confounding factors, notably non-genetic risk factors like exposure to ionizing radiation, endocrine disruptors, and medical radiation, which may cause potential residual confounding. Nevertheless, our results underscore the importance of closely monitoring patients with SLE or PBC for the development of TC, as this may inform early detection and intervention strategies.

## Data availability statement

The original contributions presented in the study are included in the article/**Supplementary Material**. Further inquiries can be directed to the corresponding author.

## Author contributions

WP: Conceptualization, Writing – original draft. BX: Formal analysis, Writing – review & editing. HZ: Formal analysis, Writing – review & editing. JD: Data curation, Writing – review & editing. XG: Data curation, Writing – review & editing. SH: Conceptualization, Writing – original draft.
