# Supplementary material for: Causal effects of autoimmune diseases on thyroid cancer: a two-sample Mendelian randomization study
Source: Front Endocrinol (Lausanne). 2024 Aug 8;15:1401458. doi: 10.3389/fendo.2024.1401458 (PMC11339619; doi:10.3389/fendo.2024.1401458)
Supplement: Supplementary file 1 [file Image_1.pdf]

Supplementary Material

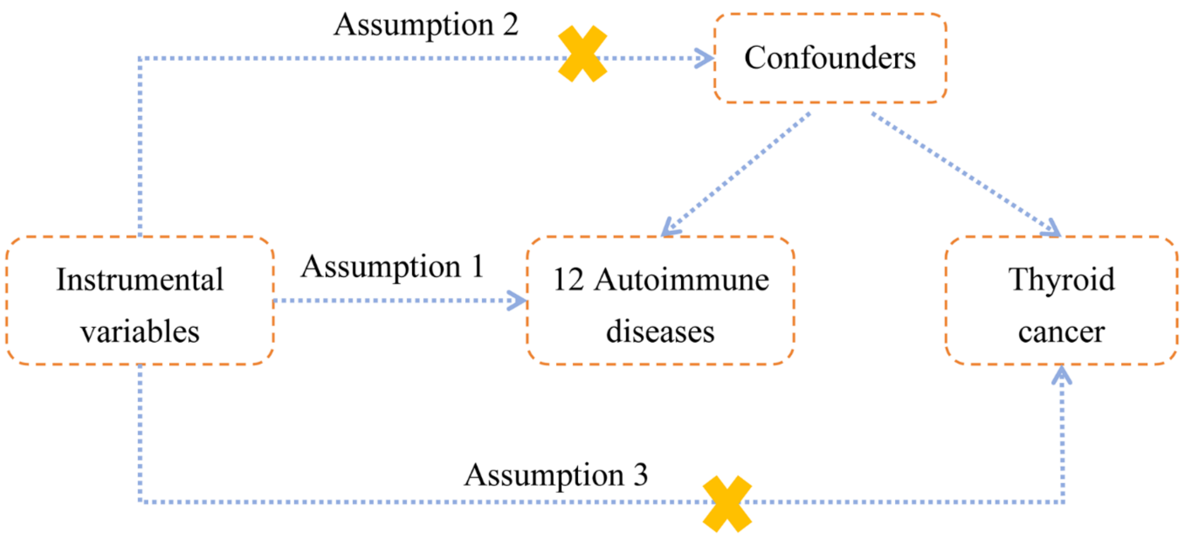

Figure S1 Assumptions of the Mendelian randomization study

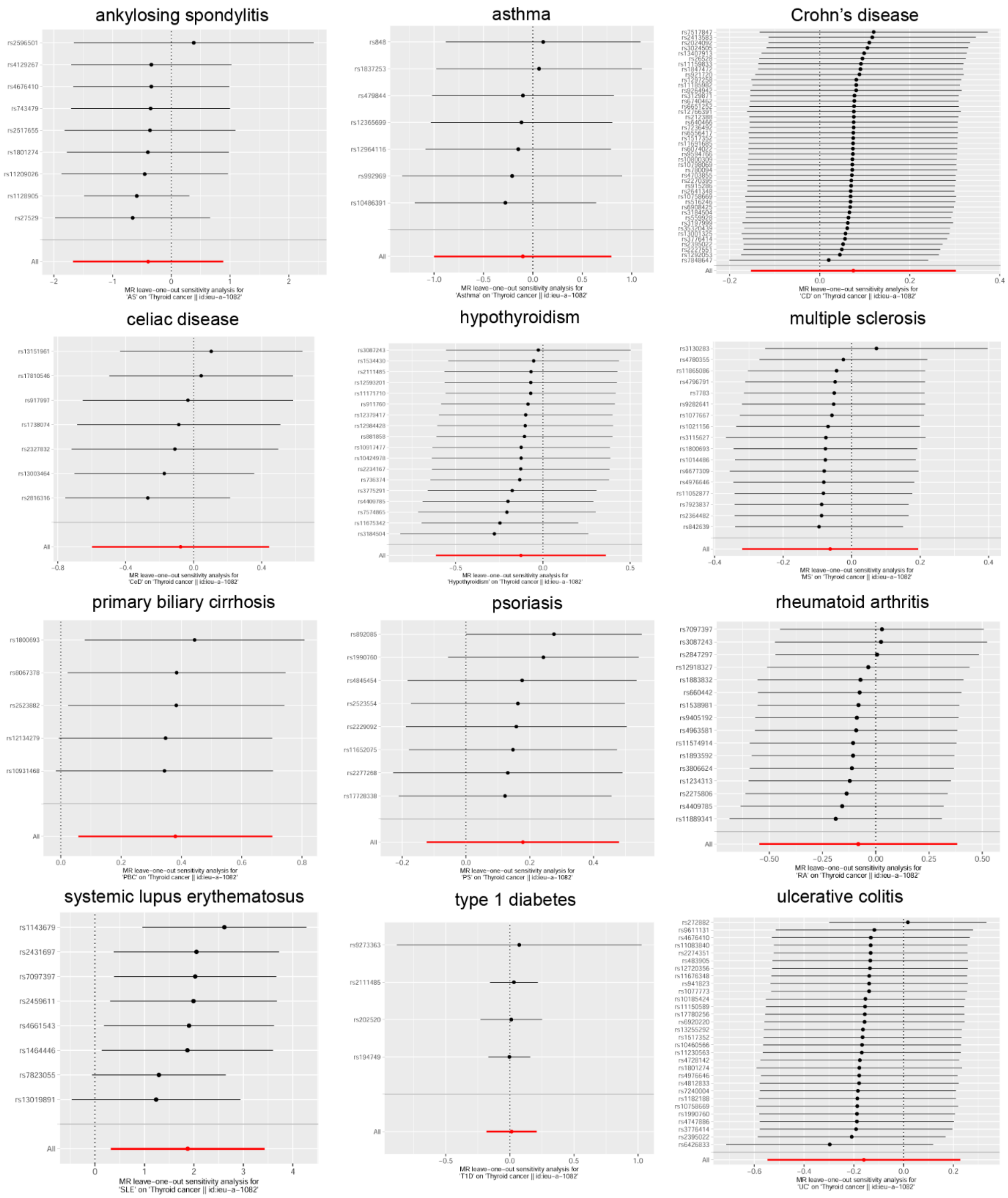

Figure S2 Leave-one-out plots for the causal relationships between AIDs and TC.

AID, autoimmune disease; TC, thyroid cancer.
